# ^18^F-FDG PET/CT in the Follow-Up of Mucosal Leishmaniasis

**DOI:** 10.4269/ajtmh.17-0660

**Published:** 2018-01

**Authors:** Raphael A. Camargo, Lázaro M. Camargo, Marcelo T. Sapienza, Carlos A. Buchpiguel, Valdir S. Amato, Felipe Francisco Tuon

**Affiliations:** 1 University of São Paulo, Medical School, São Paulo, Brazil;; 2 Veterinary Medical School, University of Cuiabá, Cuiabá, Mato Grosso, Brazil;; 3 Department of Infectious and Parasitic Diseases, University of São Paulo, Medical School, São Paulo, Brazil;; 4 Institute of Radiology, Hospital das Clínicas, University of São Paulo Medical School, São Paulo, Brazil;; 5 School of Medicine, Pontifícia Universidade Católica do Paraná, Curitiba, Brazil

Mucosal leishmaniasis (ML) is an important neglected anthropozoonosis that is endemic in most regions in Brazil.^1^ Nasal involvement changes the paranasal sinuses drainage, leading to chronic sinusitis and late facial disfigurement due to the nasal septum destruction.^2^ Facial computed tomography of patients with ML shows several structural alterations, such as erosion of the nasal septum; collapse of the nasal pyramid; thickening of the nasal cavity, nasopharynx, nasal pyramid, and soft palate; and even erosion of the nasal bone.^2^

^18^F-fluorodeoxyglucose positron emission tomography/computed tomography (^18^F-FDG–PET/CT) is an alternative imaging method for ML patients because of its accurate assessment of the glycolytic metabolism of inflammatory cells.^3,4^ This brief communication describes the use of ^18^F-FDG–PET/CT as an adjunct assessment tool in the follow-up of two patients with ML.

## Case report 1.

**Figure 1.**
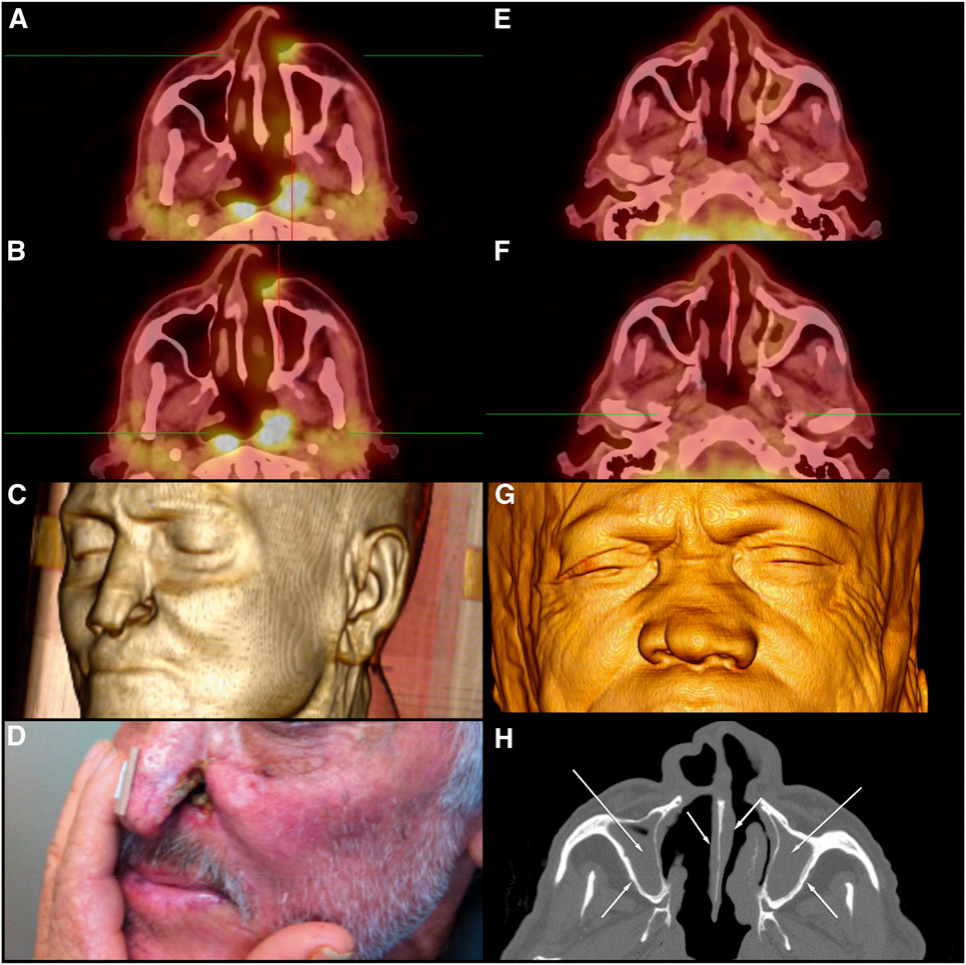
Axial ^18^F-fluorodeoxyglucose positron emission tomography/computed tomography (^18^F-FDG–PET/CT) images (**A** and **B**) of the patient from case report 1 show areas of enhanced glycolytic metabolism in a subcutaneous thickening area contiguous to the erosion of the left nasal wing (**A**) and in the asymmetric topography of the nasopharynx with obliteration of the posterolateral recess (also known as the fossa of Rosenmüller) on the left (**B**). Volume-rendered 3D image of multislice CT data (**C**) and erosion of the left nasal wing (**D**). Axial ^18^F-FDG–PET/CT images (**E** and **F**) of the patient from case report 2 show preserved glycolytic metabolism in facial structures. Volume-rendered 3D image of multislice CT data (**G**) and axial CT scans of the bone window (**H**) show diffuse thickening of the nasal wings with collapse of the nasal pyramid (**G**), and mucous thickening of the nasal fossae associated with partial opacification of the maxillary sinuses with thickening of their bony walls (osteitis-H). This figure appears in color at www.ajtmh.org.

A male patient from the northeast of Brazil reported history of ML treated with meglumine antimoniate 20 mg/kg/day during 30 days with improvement of the lesion. Eight months later the patient presented itching, pain, whitish secretion, and increased lesion size, associated with epistaxis. He was then submitted to a head and neck low-dose protocol ^18^F-FDG –PET/CT, which showed increased glycolytic metabolism in facial structures (Figure 1A and B). After biopsy, the immunohistochemical analysis was positive for *Leishmania* antigens.

## Case report 2.

A male patient, 69 years old, from the Northeast of Brazil was treated for ML in 2005. After eight annual checkups, the patient presented worsening of the nasal obstruction. A head and neck low-dose protocol ^18^F-FDG–PET/CT was requested, showing preserved glycolytic metabolism in facial structures (Figure 1E and F). Otorhinolaryngologic examination did not show mucosal alterations or signs of disease activity. CT revealed signs of chronic rhinosinusitis and facial structure alterations related to previous ML (Figure 1H). The patient was treated for chronic sinusitis and presented improvement of nasal obstruction and other symptoms. He is asymptomatic until last medical visit.

^18^F-FDG used in the PET/CT detects increased glycolytic metabolism triggered by an increased production of cytokines. This is the first report of the use of ^18^F-FDG–PET/CT in the evaluation of ML, showing a case of recurrence and another in which the examination excluded inflammatory activity despite suggestive signs.
